# The impact of frozen sections on final surgical margins in squamous cell carcinoma of the oral cavity and lips: a retrospective analysis over an 11 years period

**DOI:** 10.1186/1758-3284-3-56

**Published:** 2011-12-30

**Authors:** Stefan Gerber, Carole Gengler, Klaus W Grätz, Astrid L Kruse

**Affiliations:** 1 Department of Craniomaxillofacial and Oral Surgery, University Hospital Zurich, (Frauenklinkstrasse 24), Zurich, (CH-8091), Switzerland; 2 Department of Pathology, University Hospital Zurich, (Schmelzbergstrasse 12,) Zurich, (CH-8091), Switzerland

**Keywords:** frozen section, surgical margin, squamous cell carcinoma, oral cavity, lips

## Abstract

**Background:**

Taking intraoperative frozen sections (FS) is a widely used procedure in oncologic surgery. However so far no evidence of an association of FS analysis and premalignant changes in the surgical margin exists. Therefore, the aim of this study was to evaluate the impact of FS on different categories of the final margins of squamous cell carcinoma (SCC) of the oral cavity and lips.

**Methods:**

FS, pT-stage, grading, and tumor localization of 178 patients with SCC of the oral cavity and lips were compared by uni- and multivariate analysis in patients with positive, dysplastic and negative surgical margin status.

**Results:**

Performed on 111 patients (62.4%), intraoperative FS did not have any statistically significant influence on final margin status, independent of whether it was positive (p = 0.40), dysplastic (p = 0.70), or negative (p = 0.70). Positive surgical margins in permanent sections were significantly associated with pT4-tumors (OR 5.61, p = 0.001). The chance for negative margins in permanent sections was significantly higher in tumors located in the tongue (OR 4.70, p = 0.01).

**Conclusions:**

Our data suggests that intraoperative FS in SCC can be useful in selected cases. However it is not advisable as a routine approach.

## Background

Although no consensus exists about what constitutes a "positive" surgical margin, it is widely accepted that tumors at the inked resection margin are associated with lower survival rates [1-5]. Therefore the surgeon's primary aim is to achieve a clear surgical margin and most, but not all [6,7], centers follow this practice.

Frozen section (FS) analysis costs USD $3,123 on average per patient with an estimated cost-benefit ratio of 20:1[8]. Therefore, and because of increasing costs in the healthcare system, the diagnostic value of FS in head and neck oncological surgery was investigated recently. The impact of FS on survival and local recurrence is still controversial [9,10]. However, two studies including the same patient population [5,11] showed no effect of FS on involved surgical margins, and Ribeiro et al. stated no effect on close surgical margins [12]. So far there is no evidence of an association of FS analysis with premalignant changes in the surgical margin of permanent slides.

The aim of this study was to investigate whether FS had any effect on different categories of the final surgical margins, including carcinoma in situ/dysplasia in the margin of oral/lip squamous cell carcinoma (SCC).

## Methods

### Selection of surgical cases

Between 1998 and 2008, 374 patients with head and neck cancer were treated at the Department of Craniomaxillofacial and Oral Surgery at the University Hospital of Zurich, Switzerland. Inclusion criteria for the study were as follows: (i) patient had SCC of the oral cavity or upper/lower lips; (ii) no previous surgical excision had been performed for this tumor; and (iii) the operation was done with curative intent. Overall 196 patients had to be excluded with 63 patients showing another tumor type. Other reasons for exclusion were other localization of the tumor, palliative surgery, patient already surgically treated before, missing charts with detailed clinical and pathological information and if only dysplasia was seen in permanent histological sections when initially a SCC was anticipated. Finally, 178 patients were included in the retrospective analysis.

The study design fulfills the criteria of paragraphs 4a and b according to the guidelines of the cantonal ethics committee of Zurich and therefore is exempted from institutional review board approval.

### Data collection and definitions

Clinical variables evaluated included gender, age, tumor localization, and surgical procedure, including neck dissection. The pathological variables examined comprised pT-stage, histologic grading 1-3, histologic subtype of SCC. If FS were performed, any area that was regarded as suspicious by the surgeon was sampled. The frozen and permanent sections were evaluated by different experienced pathologists. Further, we divided the histopathological margins of permanent sections into three categories:

1) Positive margins: Involved by invasive carcinoma (incl. perineural invasion within the margin).

2) Dysplastic margins: Involved by carcinoma in situ and/or low to high grade dysplasia without invasive carcinoma in the margin.

3) Negative margins: No involvement by invasive carcinoma, carcinoma in situ, or dysplasia.

In cases with dysplastic or negative margins, the minimal distance from tumor to resection margin was recorded and divided into three categories (i) < 1 mm; (ii) 1-5 mm; and (iii) > 5 mm. All cases with positive or dysplastic margins in the permanent histological slides were examined to see if the FS showed infiltration with carcinoma (positive FS), dysplasia and/or carcinoma in situ (dysplastic FS), or if they were clear of any pathological changes (negative FS). Furthermore, it was noted if FS were performed on the same area where the histopathological changes in the permanent slide were seen.

### Data management and statistical analysis

Data were analyzed with the SPSS 17.0 using chi-squared tests for binary variables. As a baseline for regression analyses, variables were chosen on the basis of most data observed (pT1 for pT-stage, lower jaw for tumor localization, and local resection for operations) or on the basis of pathological parameters (G1 for grading). Results of the statistical analysis with p-values smaller than 5% were considered to be statistically significant. Values bigger than 5%, but smaller than 10%, were interpreted as tendencies.

## Results

A total of 178 patients (102 males and 76 females) were reviewed in this retrospective analysis. The mean age was 63.5 years (range 32 to 89). The site distribution is summarized in Table 1, with the lower jaw being the most common site encountered in 47 patients (26.4%).

**Table 1:** Characteristics of 178 patients with squamous cell carcinoma of the oral cavity and lips between 1998 - 2008

	No. of cases	%
**Patients**		
Men	102	(57.3)
Women	76	(42.7)
Mean age	63.5 years	(range 32-89)
**Tumor localization**		
Lower jaw	47	(26.4)
Tongue	39	(21.9)
Floor of mouth	34	(19.1)
Upper jaw	29	(16.3)
Upper and lower lips	15	(8.4)
Other locations	14	(7.9)
**pT-Stage**		
pT1	83	(46.6)
pT2	53	(29.8)
pT3	10	(5.6)
pT4	32	(18.0)
**Grading**		
G1	28	(15.7)
G2	108	(60.7)
G3	42	(23.6)
**Special features of tumors**		
Necrotic	1	(0.6)
Ulceration	30	(16.9)
Bone invasion	23	(12.9)
Surgical procedures*		
Local resection	68	(38.2)
Lower jaw resection	47	(26.4)
Floor of mouth/Tongue resection	34	(19.1)
Hemimaxillectomy/Upper jaw alveolar resection	27	(15.2)
Lips and other resections	8	(4.5)
**Final surgical margin status**		
Positive margin (involved by carcinoma)	41	(23.0)
Dysplastic margin	19	(10.7)
- Carcinoma in situ	1	(0.5)
- low/middle grade dysplasia	9	(5.1)
- high grade dysplasia	9	(5.1)
Negative margin	118	(66.3)
Distance carcinoma - resection margin	137	(77.0)
- < 1 mm	27	(15.2)
- 1-5 mm	86	(48.3)
- > 5 mm	24	(13.5)
**Frozen sections**		
Frozen sections done	111	(62.4)
- in cases with positive final margin (n = 41)	22	(53.7)
- in cases with dysplastic final margin (n = 19)	14	(73.7)
- in cases with negative final margin (n = 118)	75	(63.6)

* More than one surgical procedure possible

Tumor staging was as follows: pT1 in 83 (46.6%), pT2 in 53 (29.8%), pT3 in 10 (5.6%), and pT4 in 32 (18%) patients. Twenty-eight patients were identified with grade 1 histology (15.7%), 108 with grade 2 (60.7%) and 42 with grade 3 (23.6%).

### Frozen sections

FS to assess surgical margin status were performed on 111 of 178 patients (62.4%) (Table 1).

In the group of patients where FS was performed, the final surgical margin was positive in 22 cases (19.8%), being not significantly lower than in the pool of patients without FS (19 positive final margins of 67 cases without FS) (28.4%, OR 0.70, p = 0.40).

Dysplastic changes on final margins occurred more often when FS analysis was undertaken (12.6% vs. 7.5%, OR 1.26, p = 0.70). In our multivariate logistic regression analysis, the practice of using FS for achieving negative margins was not significantly associated with the margin status on permanent histological slides, independent of whether this was positive (p = 0.40), dysplastic (p = 0.70), or negative (p = 0.70) (Table 2). Univariate analysis confirmed these results.

**Table 2:** Multivariate logistic regression analysis of surgical margin status in permanent sections of oral and lip squamous cell carcinoma

	Positive surgical margin (n = 41)				Dysplastic surgical margin (n = 19)				Negative surgical margin (n = 118)			
	**No. of cases**	**Odds Ratio**	**95% CI**	**p Value**	**No. of cases**	**Odds Ratio**	**95% CI**	**p Value**	**No. of cases**	**Odds Ratio**	**95% CI**	**p Value**

**Frozen sections done**	22	0.70	0.31 - 1.61	0.40	14	1.26	0.39 - 4.07	0.70	75	1.15	0.56 - 2.37	0.70
**pT-Stage**												
pT1*	10	-	-	-	9	-	-	-	64	-	-	-
pT2	10	1.72	0.61 - 4.83	0,31	9	1.39	0.47 - 4.09	0.55	34	0.57	0.25 - 1.29	0.18
pT3	4	4.44	0.97 - 20.35	0.055	1	0.77	0.08 - 7.76	0.83	5	0.33	0.08 - 1.38	0.13
pT4	17	5.61	1.98 - 15.87	0.001	0	-	-	-	15	0.42	0.16 - 1-07	0.07
**Grading**												
G1*	5	-	-	-	2	-	-	-	21	-	-	-
G2	27	1.43	0.41 - 4.91	0.57	12	1.35	0.26 - 7.07	0.72	69	0.76	0.27 - 2.14	0.61
G3	9	1.03	0.25 - 4.23	0.97	5	1.21	0.19 - 7.68	0.84	28	1.02	0.32 - 3.32	0.97
**Tumor localization**												
Lower jaw*	19	-	-	-	5	-	-	-	23	-	-	-
Floor of mouth	7	0.49	0.16 - 1.47	0.20	7	1.44	0.37 - 5.57	0.59	20	1.35	0.53 - 3.47	0.53
Tongue	3	0.21	0.05 - 0.84	0.03	3	0.39	0.08 - 1.83	0.23	33	4.70	1.55 - 14.23	0.01
Upper and lower lips	2	0.42	0.08 - 2.30	0.32	1	0.38	0.04 - 3.70	0.40	12	3.07	0.72 - 13.11	0.14
Upper jaw	8	0.77	0.25 - 2.35	0.64	1	0.20	0.02 - 1.88	0.16	20	2.09	0.75 - 5.85	0.16
Other locations	2	0.57	0.10 - 3.14	0.52	2	0.73	0.12 - 4.52	0.73	10	1.80	0.46 - 7.06	0.40

* Baseline

The proportion of FS performed ranges from 15 of 32 cases (46.9%) in pT4- tumors to 8 of 10 cases (80%) in pT3-tumors and a multivariate logistic regression analysis confirmed that FS were significantly more often taken from pT2-tumors (OR 2.53, p = 0.03) compared to pT1-tumors. Tumor localization did not seem to have any statistically significant influence on the decision to take FS.

To focus on tumors with positive or dysplastic final surgical margins, FS were more frequently performed in cases with dysplastic than with positive margins (53.7% vs. 73.7%). In the group of tumors with final positive surgical margins, FS analysis was undertaken in 22 of 41 cases (53.7%), of which ten (45.5%) showed invasive carcinoma or dysplasia in FS. In one half of these 10 cases, further FS were taken from the same site until a negative intraoperative result was obtained. In the other half, no further FS were taken, and the tumor was excised with a wide margin at this crucial site. In all of the 10 cases with positive or dysplastic FS and final positive surgical margins, the sites from which FS were taken and the areas where the final surgical margin was positive were not identical. In 5 of the other 12 patients, FS were performed on the same site where the final surgical margin was positive, which accounts for 22.7% of all patients with positive final surgical margin and positive, dysplastic or negative FS.

In 14 out of 19 patients (73.7%) with dysplastic final surgical margin status, FS were performed. Half of these 14 patients showed positive or dysplastic FS; in one patient, the FS was repeated until a negative result was achieved; and in the other 6 cases, tumors were excised with a wider margin, without a second FS analysis undertaken. However, in 5 of these 6 tumors, dysplastic final surgical margins were reported as being located at the same site as the FS. In 11 of 14 patients (76.8%), FS were located at the same site where the final surgical margin showed dysplasia/carcinoma in situ. Overall, in 16 out of 36 patients (44.4%) with FS and positive or dysplastic final margins, the location was identical according to the pathology report.

### Final Surgical margin status

In 41 of 178 patients (23%), the histological margins of the permanent slides were involved with invasive carcinoma and were considered to be positive. Nineteen patients (10.7%) showed either low/middle grade dysplasia (9 patients) or high grade dysplasia/carcinoma in situ (10 patients) in the final surgical margin and were considered to have dysplastic margins for data analysis. However, the majority of patients had negative surgical margins (118 patients, 66.3%) (Table 1).

**Figure 1:**
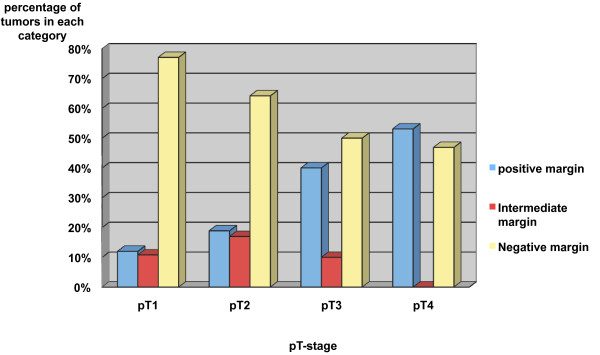
**Margin status in relation to pT-stage**.

The chance of a positive final surgical margin is significantly increased by the factor 5 in pT4-stage tumors compared to pT1-tumors (OR 5.61, p = 0,001). Showing the same tendency are pT3-stage tumors (OR 4.44, p = 0,055) (Table 2). Consequently, histologically proven bone invasion, which was reported in 23 of 178 cases (12.9%), showed a significant correlation to positive surgical margins in permanent slides (OR 4.47, p < 0.001). Figure 1 demonstrates the increasing ratio of positive final margins with increasing pT-stage, and the inverse correlation with negative surgical margins. Regarding the tumor site, the tongue is rarely associated with positive surgical margins in permanent slides (OR 0.21, p = 0,03); consequently, tumors located at this site have a 4.7-fold increased chance of a negative surgical margin (OR 4.70, p = 0.01) (Table 2). This could be confirmed in a univariate logistic regression analysis demonstrating significantly more frequent negative final surgical margins after floor-of-mouth resection, including tongue resection, compared to local resection (OR 4.19, p = 0.01). In addition, floor of mouth and tongue resection (OR 0.17, p = 0.09) and hemimaxillectomy including upper alveolar resection (OR 0.14, p = 0.06) both tend to be associated with dysplastic final surgical margins in permanent slides (Table 3).

**Table 3:** Univariate logistic regression analysis about different surgical procedures and the surgical margin status

	Positive surgical margin (n = 41)				Dysplastic surgical margin (n = 19)				Negative surgical margin (n = 118)			
	**No. of cases**	**Odds Ratio**	**95% CI**	**p Value**	**No. of cases**	**Odds Ratio**	**95% CI**	**p Value**	**No. of cases**	**Odds Ratio**	**95% CI**	**p Value**

**Operations**												
Local resection*	16	-	-	-	14	-	-	-	38	-	-	-
Hemimaxillectomy/Upper jaw alveolar resection	6	0.93	0.33 - 2.74	0.93	1	0.17	0.02 - 1.33	0.09	20	2.03	0.77 - 5.37	0.15
Floor of mouth/Tongue resection	4	0.42	0.13 - 1.32	0.14	1	0.14	0.02 - 1.08	0.06	29	4.19	1.48 - 11.83	0.01
Lower jaw resection	16	1.92	0.87 - 4.25	0.11	4	0.44	0.14 - 1.46	0.18	27	0.83	0.40 - 1.72	0.61
Other operations	1	0.45	0.05 - 3.84	0.46	0	-	-	-	7	5.06	0.60 - 42.91	0.14

* Baseline

* Basel

### Distance from tumor to resection margin

Out of 137 surgical specimens with dysplastic or negative margin status in permanent slides, 110 tumors (80.3%) were located at least 1 mm away from the surgical margin with a distance between 1 and 5 mm most often reported (in 86 of 137 cases, 62.8%) (Table 1).

**Figure 2:**
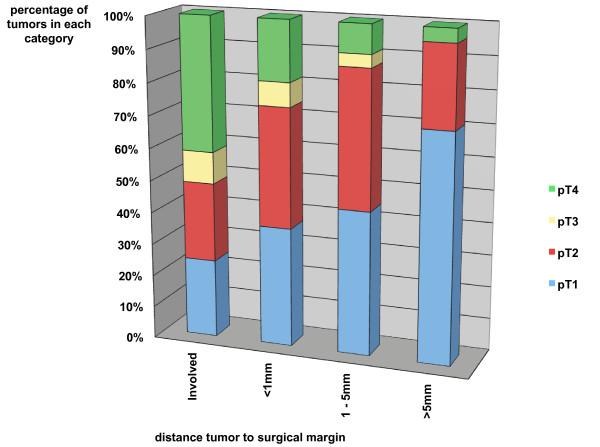
**Distance [mm] from carcinoma to surgical margin according to pT-stage**.

The distance from tumor to resection margin is significantly influenced by pT-stage (pT2 p = 0.03, pT3 p = 0.01, pT4 p < 0.001) and by tumors located in the tongue (p = 0.01). At this site, 22 of 39 tumors (56.4%) were located within 1-5 mm from the final margin and only 3 surgical specimens (7.7%) showed infiltration of the margin by invasive carcinoma. Figure 2 demonstrates the correlation of pT-stage with distance from tumor to final resection margin. With increasing distance to surgical margin, the proportion of pT1-tumors increases from 24.4% with positive margins to 70.8% with a clear margin of more than 5 mm. The inverse correlation is illustrated for pT4-tumors with a decrease from 41.5% to 4.2%.

## Discussion

All together 178 patients were studied, presenting with SCC of the oral cavity or lips between 1998 and 2008, who were surgically treated with a curative intention. In 111 (62.5%) patients, intraoperative FS for margin evaluation were performed. The practice of taking FS did not have any statistically significant influence on the final margin status of the surgical specimen, irrespective of whether the margin was involved by invasive carcinoma or by dysplasia/carcinoma in situ. Furthermore, the data support that FS may be negative, although taken from the same area where the final surgical margin was classified as positive or dysplastic. However, final surgical margin status was significantly associated with two factors: tumor localization and pT-stage. Tumors located in the tongue led more often to negative margins, and pT4-tumors had 5 times as many positive margins as pT1-tumors. In negative or dysplastic final surgical margins, the distance from tumor to resection margin increased with pT-stage and showed a significant correlation to tumors located in the tongue.

The influence of FS on final surgical margin status had not been assessed until lately. Binahmed et al. [5] examined the clinical significance of positive surgical margins in permanent slides in a cohort of 425 patients with oral SCC. The diagnosis was biopsy-proven, and patients had been previously untreated. Intraoperative FS, performed on 52.9% of the patients, were associated neither with involved nor with clear final margins. However, there are no p-values available in this paper, and it is not clear if uni- or multivariate analysis was used. Similar results were published by Nason et al. [11], who investigated the same patient cohort as Binahmed et al [5]. They performed a subgroup-analysis with a remaining cohort of 277 patients, recording the width of the clear margin. Like in both studies, we also could not find any significant association of FS and margin status, independent of whether the final margin was positive, dysplastic, or negative.

Byers et al. [13] reviewed 216 patients with SCC of the head and neck, who underwent surgical treatment including FS analysis. Of these tumors, 67% were adequately excised through the surgeon's judgement. Moreover, in the current study, in 36 of 60 patients (60%), positive or dysplastic final surgical margins went undetected in spite of using FS. In more recent studies this figure for undetected positive surgical margins ranges between 15.4% and 83.3%[8,10,12,14], raising the question about the diagnostic value of FS.

In only 16 of the 36 patients (44.4%), FS were located in the same area where the final surgical margin showed pathological changes. This finding demonstrates one of the main limitations of FS: the sampling [6,15]. Tumors are 3-dimensional structures, and it is not practicable to evaluate the whole surgical margin by means of FS [1]. This highlights the importance of sampling the crucial sites from which FS should be taken. So far no reliable test exists to detect in-vivo where the tumor front is located.

After an initially positive FS, a second FS was done in 3 of 17 patients (17.6%) until it was negative. In one of these 3 patients, dysplasia was found in the margin of the permanent section at the same site. The procedure of doing multiple FS at the same site leads to another source of bias: the relocation by the surgeon. Kerawala et al. [16] showed that the mean error in relocating the site of FS in oropharyngeal cancer is 9 mm for samples at the mucosal margin and 12 mm for samples placed deep into the tumor.

Meier, Oliver, and Varvares [7] found in their survey of 1500 members of the International American Head and Neck Society, that 97% use FS for margin evaluation in the oral cavity and pharynx, with 76% taking those from the surgical bed and 14% from the resected surgical specimen. However, no explicit evidence exists on whether FS should be taken at the surgical bed or from the specimen [7,14].

Positive surgical margins, one of the most important prognostic factors [17], are 1.7 times more likely to be encountered in oral carcinoma than in other head and neck tumors [1]. In the present study, 23% of all the patients had a positive final surgical margin in permanent sections. This finding is similar to the results of other studies that reported ratios between 4.5% and 52.9%[1-5,11,12,14,18,19].

Tumors from the tongue were associated more frequently with negative surgical margin status in our study, a fact that has been reported before [18]. This may be explained by the fact that the circumference of the tumor is easily palpable and that less anatomical limits give a good surgical access to the tumor site.

The present study has a number of limitations. It did not differentiate between patients with and without prior radiotherapy. Preoperative radiotherapy is often associated with extensive fibrosis and inflammatory reaction, which makes the surgical resection and the histopathological assessment of FS more difficult [20]

## Conclusion

In conclusion, the present results show that the diagnostic value of intraoperative FS for margin assessment in SCC of the oral cavity and lips is limited. Even in cases with reported negative FS, the final surgical margin of immediately adjacent tissue can be positive in the final histological evaluation. This highlights the fact that FS are only as good as the sampling is [12], although the accuracy of FS itself was previously reported to be over 98%[8,12].

The practice of routinely doing FS in SCC of the oral cavity and the lips is questionable. It does not seem to significantly influence the outcome of final surgical margin status and in some situations it may even mislead the surgeon. The necessity of taking FS should be evaluated case by case. The specific situations in which FS are necessary and what is the most useful sampling protocol may be the subject of further studies.

## Competing interests

The authors declare that they have no competing interests.

## Authors' contributions

SG and AK conceived of the study, participated in its design and coordination. SG and AK drafted and designed the manuscript and contributed equally to this work. CG made substantial contribution to data acquisition and conception of manuscript. CG and KG were involved in revising the manuscript. All authors read and approved the final manuscript.
